# Supplementary material for: Sensory cortex is optimized for prediction of future input
Source: eLife. 2018 Jun 18;7:e31557. doi: 10.7554/eLife.31557 (PMC6108826; doi:10.7554/eLife.31557)
Supplement: Transparent reporting form [file elife-31557-transrepform.docx]

***eLife’s* transparent reporting form**

We encourage authors to provide detailed information *within their submission* to facilitate the interpretation and replication of experiments. Authors can upload supporting documentation to indicate the use of appropriate reporting guidelines for health-related research (see [EQUATOR Network](http://www.equator-network.org/%20)), life science research (see the [BioSharing Information Resource](https://biosharing.org/)), or the [ARRIVE guidelines](http://www.plosbiology.org/article/info:doi/10.1371/journal.pbio.1000412) for reporting work involving animal research. Where applicable, authors should refer to any relevant reporting standards documents in this form.

If you have any questions, please consult our Journal Policies and/or contact us: [editorial@elifesciences.org](mailto:editorial@elifesciences.org).

**Sample-size estimation**

- You should state whether an appropriate sample size was computed when the study was being designed
- You should state the statistical method of sample size computation and any required assumptions
- If no explicit power analysis was used, you should describe how you decided what sample (replicate) size (number) to use

Please outline where this information can be found within the submission (e.g., sections or figure legends), or explain why this information doesn’t apply to your submission:

This was a largely theoretical study and the results of the modelling were then compared to previously published data. As such, the sample size was generally limited according to the requirements of the previously published studies from which the data were taken. Nevertheless, for the statistical tests done, the sample sizes used substantially exceed the minimum sample sizes required for the given statistical tests. In all cases where these are performed, the sample size and other relevant statistics are reported.

**Replicates**

- You should report how often each experiment was performed
- You should include a definition of biological versus technical replication
- The data obtained should be provided and sufficient information should be provided to indicate the number of independent biological and/or technical replicates
- If you encountered any outliers, you should describe how these were handled
- Criteria for exclusion/inclusion of data should be clearly stated
- High-throughput sequence data should be uploaded before submission, with a private link for reviewers provided (these are available from both GEO and ArrayExpress)

Please outline where this information can be found within the submission (e.g., sections or figure legends), or explain why this information doesn’t apply to your submission:

For all the datasets used, the number of replications is given in the text. In the case of the datasets we used, a biological replicate is a new neuron whose response to stimulus is recorded from. A technical replicate is a repeat of the same stimulus played to the cell. We make it clear in the manuscript where one or the other is used, denoting biological replicates by ‘neurons’ or ‘units’ and technical replicates by ‘repeats’ and give the number of replicates used where relevant. In the Methods section, we state very explicitly, the criteria used for data exclusion and indicate the number of data points included and excluded in each case. These were the only criteria used to exclude data and in no analysis was data excluded simply for being an outlier. In Fig 7d a few data points (4/473) for the temporal prediction model are not shown (but were included in all numerical analyses). The same is true for Fig 6 and Fig 6-Fig Supplement 1, where in each case a couple of data points (2/100) for the sparse coding model are not shown. These points were so far from the rest of the distribution that to show them would shrink the distribution for the real data to a size that its form would not be clearly observable.

**Statistical reporting**

- Statistical analysis methods should be described and justified
- Raw data should be presented in figures whenever informative to do so (typically when N per group is less than 10)
- For each experiment, you should identify the statistical tests used, exact values of N, definitions of center, methods of multiple test correction, and dispersion and precision measures (e.g., mean, median, SD, SEM, confidence intervals; and, for the major substantive results, a measure of effect size (e.g., Pearson's r, Cohen's d)
- Report exact p-values wherever possible alongside the summary statistics and 95% confidence intervals. These should be reported for all key questions and not only when the p-value is less than 0.05.

Please outline where this information can be found within the submission (e.g., sections or figure legends), or explain why this information doesn’t apply to your submission:

The statistical analyses methods we used are: receptive field estimation through multivariate regression using L1 regularisation (LASSO). This is a state of the art technique for receptive field estimation that has been used in our own^1^ and others^2^, previous work; multidimensional scaling to produce a 2D representation of a high dimensional dataset, which is the standard technique for this analysis; Kolmogorov-Smirnov statistic, a standard measure for distribution comparisons; Pearson’s correlation coefficient, which is a standard measure and test of correlation.

We present the data at the most informative level for the questions we ask, which in this case is the receptive field. We do not focus on spike trains or raw electrical traces as this is primarily a theoretical paper and the details of the experimental data used are already published elsewhere. We do show example images and spectrograms of the input stimuli we use for our models (Fig 1) and we also show every single receptive field of substantially responsive units for both the visual and the auditory models at the optimal settings and for the auditory data used for comparison.

All statistical tests are identified in the results section on pages 14, 16 and 17 together with P and N values.

References:

1.	Harper, N. S. *et al.* Network Receptive Field Modeling Reveals Extensive Integration and Multi-feature Selectivity in Auditory Cortical Neurons. *PLOS Comput. Biol.* **12,** e1005113 (2016).

2.	David, S. V., Mesgarani, N. & Shamma, S. A. Estimating sparse spectro-temporal receptive fields with natural stimuli. *Netw. Comput. Neural Syst.* **18,** 191–212 (2007).

(For large datasets, or papers with a very large number of statistical tests, you may upload a single table file with tests, Ns, etc., with reference to sections in the manuscript.)

**Group allocation**

- Indicate how samples were allocated into experimental groups (in the case of clinical studies, please specify allocation to treatment method); if randomization was used, please also state if restricted randomization was applied
- Indicate if masking was used during group allocation, data collection and/or data analysis

Please outline where this information can be found within the submission (e.g., sections or figure legends), or explain why this information doesn’t apply to your submission:

This is not applicable to our study.

**Additional data files (“source data”)**

- We encourage you to upload relevant additional data files, such as numerical data that are represented as a graph in a figure, or as a summary table
- Where provided, these should be in the most useful format, and they can be uploaded as “Source data” files linked to a main figure or table
- Include model definition files including the full list of parameters used
- Include code used for data analysis (e.g., R, MatLab)
- Avoid stating that data files are “available upon request”

Please indicate the figures or tables for which source data files have been provided:

All custom code used in this study was implemented in MATLAB and Python. We have uploaded the code to a public Github repository^92^. The raw auditory experimental data is available at  <https://osf.io/ayw2p/>. The movies and sounds used for training the models are all publicly available at the websites detailed in the Methods.
